# The efficacy of oxytocin gel in postmenopausal women with vaginal atrophy: an updated systematic review and meta-analysis

**DOI:** 10.1186/s12905-023-02645-0

**Published:** 2023-09-16

**Authors:** Ramadan Abdelmoez Farahat, Hazem Mohamed Salamah, Abdelrahman Mahmoud, Esraa Hamouda, Mahmoud Hashemy, Heba Hamouda, Ali Samir, Imane Chenfouh, Ahmed Marey, Dina M. Awad, Elsayed Farag, Mohamed Abd-Elgawad, Elsayed Eldesouky

**Affiliations:** 1 Faculty of Medicine, Kafrelsheikh University, Kafrelsheikh, Egypt; 2 Faculty of Medicine, Zagazig University, Zagazig, 44519 Egypt; 3 Faculty of Medicine, Minia University, Minia, Egypt; 4 Faculty of Medicine, Menoufia University, Menoufia, Egypt; 5 Faculty of Medicine, Cairo University, Giza, Egypt; 6 Faculty of Medicine, Assiut University, Assiut, Egypt; 7 Faculty of Medicine and Pharmacy, Oujda, Oujda-Angad Morocco; 8 Faculty of Medicine, Alexandria University, Alexandria, Egypt; 9 Department of Obstetrics and Gynecology, Faculty of Medicine, Alazhar University, Cairo, Egypt; 10 Faculty of Medicine, Fayoum University, Fayoum, Egypt

**Keywords:** Oxytocin, Postmenopausal, Post-menopause, Atrophy, GSM, Genitourinary, Maturation, Vaginal

## Abstract

**Background:**

Genitourinary syndrome of menopause (GSM) is a common and disturbing issue in the postmenopausal period. Unlike vasomotor symptoms, it has a progressive trend. Our study aims to evaluate the efficacy and safety of oxytocin gel versus placebo gel in postmenopausal women with GSM.

**Methods:**

A systematic review and meta-analysis synthesizing randomized controlled trials (RCTs) from Web of Science, SCOPUS, PubMed, and Cochrane Central Register of Controlled Trials databases on January 18, 2023. Keywords such as “oxytocin,“ “intravaginal,“ “vaginal,“ “atrophic,“ and “atrophy” were used. We used Review Manager (RevMan) version 5.4 in our analysis. We used the risk ratio (RR) for dichotomous outcomes and the mean difference (MD) for continuous outcomes; both were presented with the corresponding 95% confidence interval (CI) and were calculated with the Mantel-Haenszel or inverse variance statistical method. Cochrane’s Q test and the I^2^ statistic were used as measures of statistical inconsistency and heterogeneity. The Cochrane Risk of Bias Tool for RCTs was used for the quality assessment of the included studies.

**Results:**

Seven studies with 631 patients were included. Regarding the maturation index, there was a statistically insignificant increase in the oxytocin arm (MD = 12.34, 95% CI (-12.52-37.19), *P* = 0.33). Clinically assessed vaginal atrophy showed a statistically significant reduction in the oxytocin group (RR = 0.32, 95% CI (0.23 − 0.10), *P* < 0.00001). For dyspareunia, vaginal pH, and histological evaluation of vaginal atrophy, there was a statistically insignificant difference between the two groups (RR = 1.02, 95% CI (0.82–1.27), *P* = 0.84), (MD = -0.74, 95% CI (-1.58-0.10), *P* = 0.08), and (MD = -0.38, 95% CI (-0.82-0.06), *P* = 0.09), respectively. There was no significant difference in the safety profile between the two groups as measured by endometrial thickness (MD = 0.00, 95% CI (-0.23-0.23), *P* = 0.99).

**Conclusions:**

Although oxytocin has been proposed as a viable alternative to estrogen in the treatment of GSM, our findings show the opposite. Larger, high-quality RCTs are needed to confirm or refute our results.

**Trial registration:**

PROSPERO registration number CRD42022334357.

**Supplementary Information:**

The online version contains supplementary material available at 10.1186/s12905-023-02645-0.

## Background

Menopause is defined as the spontaneous stoppage of menses for 12 months [1]. After menopause, estrogen levels decrease markedly due to the loss of the ovarian ability to produce it. Estrogen deficiency is accompanied by broad systemic, histological, and cytological changes. These changes can cause various signs and symptoms like vasomotor hot flushes, sleep disturbances, mood changes, loss of skin elasticity, and Genitourinary syndrome of menopause (GSM), reducing the quality of life (QOL) in the affected postmenopausal women [2, 3]. The genitourinary syndrome of menopause is a fairly new concept describing the decadence of the vaginal and urethral tissue, causing various signs and symptoms, including not only dryness and itching in the genital area but also painful sexual life and lower urinary tract symptoms like urgency, dysuria, and recurrent urinary tract infections [4, 5]. The introduction of the new name was to overcome the restrictive vision of the previously used terms, such as vulvovaginal atrophy and atrophic vaginitis, which did not cover the whole scope of the subject, discarding the urinary symptoms and not linking these symptoms to estrogen decline after menopause [5].

GSM is a common health issue affecting up to 70% of postmenopausal women [6]. Within 6 years of menopause, 84% of menopausal women reported GSM [7]. In addition, 75% of women with GSM felt that GSM had a negative impact on their lives [8]. Furthermore, GSM symptom severity was associated with a worse quality of life [9], and unlike vasomotor symptoms, GSM is a progressive disease that rarely improves without treatment.

Vaginally administered estrogen is the most studied intervention and the mainline of treatment to antagonize urogenital aging and improve the QOL in this population of patients [10]. Nonetheless, the safety profile of estrogen is controversial in the long run due to the fear of increasing the risk of certain types of cancer and thromboembolic events; we need to mention that this quarrel is due to limited data [11]. New, non-estrogenic treatment options are imperative to provide an alternative to estrogen, especially when there is a concern about developing estrogen-related cancers. Oxytocin is a new modality to counteract vaginal atrophy studied in preclinical and clinical trials [12–19].

Oxytocin is a pituitary hormone formed of nine amino acids in the hypothalamus’s supraoptic and paraventricular neurons, stored in the posterior pituitary gland, and released directly into the blood via exocytosis [20]. The major role of oxytocin was confined to the obstetric field in inducing labor and reducing postpartum hemorrhage. However, research has shed light on other facets of oxytocin and widened the scope of its clinical applications [21].

Intravaginally applied oxytocin preparations work directly on its receptors in the lower genitourinary tract, thus avoiding or at least reducing its unwanted systemic actions [22]. Oxytocin can stimulate mitotic divisions by enhancing the production of multiple growth factors, mucosal blood flow, and wound healing, thus antagonizing urogenital aging [12].

There is a previously published meta-analysis on the same topic [13], but its main defect was its limited sample size of 388 participants. It also missed reporting important outcomes such as vaginal pH, clinically assessed vaginal atrophy, and dyspareunia. Furthermore, it did not address the high heterogeneity between the studies. Our objective is to update the previous study [13] with a further one included study increasing the sample size to 545 participants, representing about 40% of its sample size. We also addressed the missing outcomes and the high heterogeneity. Therefore, we have conducted a more comprehensive and updated meta-analysis to assess the efficacy and safety of intravaginal oxytocin gel in postmenopausal women with vaginal atrophy.

## Methods

The PRISMA guidelines for reporting systematic reviews and meta-analyses of randomized controlled trials (RCTs) were used as guidance by the authors [23]. This systematic review was registered within the International Prospective Register of Systematic Reviews (PROSPERO), registration identifier CRD42022334357.

### Eligibility criteria

Only randomized control trials comparing oxytocin preparations with a placebo for relieving vaginal atrophy were included in this study. We included RCTs only as they are the highest level of evidence to establish causal associations. There were no restrictions concerning race, country, or time of publication. We included the studies based on the PICO criteria: patients, intervention, control, and outcomes. The participants of interest were postmenopausal women, who were defined as women aged more than 40 years with spontaneous or surgical menopause, who had their last menstruation 12 months before the study, or who had a serum follicle-stimulating hormone (FSH) level ≥ 40 IU/L. We used a broad definition of postmenopause to increase our sample size. The intervention was intravaginal oxytocin forms (such as Pitocin, Syntocinon, and Vagitocin). The comparator was placebo. To be included, studies must have measured and reported our outcomes of interest. No limits concerning the time of follow-up were used.

Our exclusion criteria were: non-randomized studies, animal studies, conference abstracts, non-English papers, and single-arm studies. We excluded studies that compared oxytocin to active control, as our objective was to establish if intravaginal oxytocin has any beneficial effect in women with GSM and not to compare its efficacy to other active treatments. Outcomes and study results that had different measurement units or were reported in a way that could not be pooled together in a meta-analysis were excluded.

### Information sources

Relevant articles were identified through a comprehensive search on PubMed, Web of Science, Cochrane, and Scopus databases from inception to January 18, 2023. The reference lists of the eligible papers were also searched to find other relevant studies.

### Search strategy

A search was carried out for randomized controlled trial studies published in PubMed, Web of Science, Scopus, and Cochrane with no language and time restrictions using the following query: (Oxytocin OR Pitocin OR Syntocinon OR Vagitocin OR Duratocin OR Carbetocin) AND ((Intravaginal OR vaginal OR Vulvovaginal OR dyspareunia OR urogenital OR uro-genital OR Vulvar OR vulvo‐vaginal) AND (atrophic OR atrophy OR vaginitides OR vaginitis OR atrophies OR lubrication OR dryness)). The full search strategy for each database is shown in Additional file 1.

### Selection process

Using Endnote, all records were pooled. The data were exported to an Excel sheet, and then this Excel sheet was submitted through two phases to find eligible studies. First is the title and abstract screening phase, and articles that pass this phase move on to the full-text screening phase. Worth noting is that each article’s eligibility in each phase was evaluated by two authors independently, then discussed. Any conflicts were resolved by a third senior author.

### Data collection process

The lead author prepared formatted Excel sheets including baseline data and study characteristics, as well as ROB assessment and outcomes of interest. Data from each study were extracted by two authors independently, then discussed. Any conflicts were resolved by a third senior author. Any incomplete or incompatible data have been dealt with using methods recommended in the Cochrane Handbook [24].

### Data items (outcomes)

The primary outcome was the cytological assessment of vaginal atrophy (Vaginal Maturation Index), which is the relative proportion of superficial cells, intermediate cells, and parabasal cells. Maturation index = Superficial cells (%) + 0.5 × Intermediate cells (%). Secondary outcomes included laboratory indicators of vaginal atrophy, such as vaginal pH and histological assessment of vaginal atrophy; clinically objective indicator of vaginal atrophy assessed by the physical examination of the vulva and vagina or by colposcopic examination, dyspareunia, which is a subjective indicator of vaginal atrophy; and the safety profile measured by the endometrial thickness.

### Data items (other variables)

Two review authors extracted the characteristics of the studies and baseline data. Study characteristics included study ID, name and dose of the intervention and control, sample size, study design, follow-up duration, inclusion and exclusion criteria, and the results. Baseline data included the age, body mass index, and years after menopause.

### Study risk of bias assessment

The quality of the included studies was assessed with the Cochrane Risk of Bias Tool for RCTs [25]. The quality of each study was evaluated by two authors independently, then discussed. Any conflicts were resolved by a third senior author. A funnel plot was used to assess publication bias.

The tool consists of the following domains: random sequence generation (selection bias), allocation sequence concealment (selection bias), blinding of participants and personnel (performance bias), blinding of outcome assessors (detection bias), incomplete outcome data (attrition bias), selective outcome reporting (reporting bias), and other bias. Author judgments fall into three categories: low, unclear, or high risk of bias for each domain.

According to Egger’s funnel-plot-based methods, we could not assess the risk of publication bias due to the small number of included studies [26].

### Effect measures and statistical synthesis

The statistical analyses were used according to the guidelines in the Cochrane Handbook for the systematic review of interventions [24]. We used Review Manager (RevMan) version 5.4 [27]. All data were collected as means ± standard deviation (SD), or event and total for continuous and dichotomous outcomes, respectively. The continuous outcome data of maturation index, vaginal pH, histological evaluation of vaginal atrophy, and endometrial thickness were measured using the inverse variance statistic method and reported as mean differences with a 95% confidence interval (CI), and we used the Mantel-Haensze equation to calculate the pooled RR and 95% CI for dichotomous variables, such as clinically assessed vaginal atrophy and dyspareunia. A *P* value of < 0.05 was considered statistically significant. We used Cochrane’s Q test and the I^2^ statistic as measures of statistical inconsistency and heterogeneity. A random-effects model was used if there was significant heterogeneity as determined by a *P* value less than 0.05 or I^2^ higher than 60%; otherwise, we used a fixed-effects model. We used the leave-one-out method to solve and detect the cause of heterogeneity.

## Results

### Search results

**Fig. 1:**
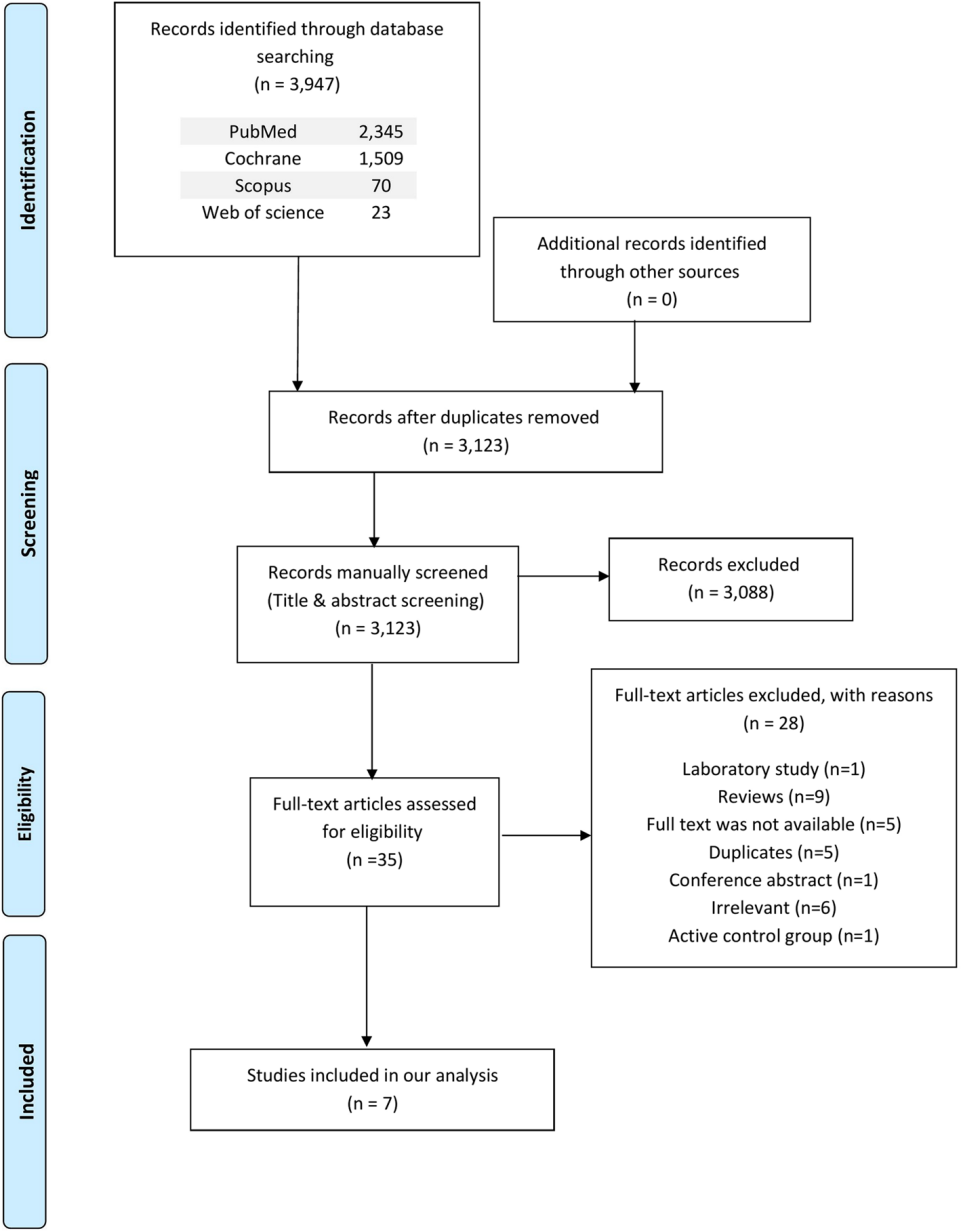
A flowchart shows the detailed process of the search strategy and study selection

Our search, carried out on January 18, 2023, retrieved 3,947 articles: 2,345 from PubMed, 1,509 from Cochrane, 70 from Scopus, and 23 from Web of Science. After the removal of duplicates, the total number was 3123. Following title and abstract screening, only 35 papers were eligible for full-text screening. Finally, seven papers were found to be eligible for the final analysis. The PRISMA flow diagram is shown in Fig. 1.

### Study characteristics

The seven included studies [14–19, 28] were double-blinded, randomized, placebo-controlled trials published between 2011 and 2020, with different follow-up periods ranging from 7 to 14 weeks in AL-saqi et al. studies [18, 19], one month in Torky et al. [15], eight weeks in Abedi et al. [17] and Zohrabi et al. [28], and 1,12 weeks in Jonasson et al. studies [14, 16]. Four trials were conducted in Sweden [14, 16, 18, 19], two in Iran [17, 28], and another one in Egypt [15]. All included studies used intravaginal oxytocin gel versus placebo, with different oxytocin doses ranging from 100 to 400 and 600 IU. Table 1 presents the study summary of the included studies.

**Table 1:** Summary of included studies

First author (year)	Study design	Sample size	Dose of oxytocin	Control group	Follow up duration	Inclusion criteria	Exclusion criteria	Results
Jonasson et al. (2011) [14]	Double-blinded, randomized pilot study.	20	1 mg.	Placebo.	7 days.	Postmenopausal women (at least two years after menopause) who were suffering from symptoms of vaginal atrophy and had not used any estrogen or other hormonal treatments (systemic or topical) during a four-week period prior to the trial.	Women who have an endocrine disease and any other serious illnesses.	Oxytocin normalizes the morphological appearance of the vaginal mucosa and promotes the restoration of the vaginal epithelium.
Al-saqi et al. (2015) [18]	Double-blinded, randomized controlled trial.	64	400 and 100 IU.	Placebo.	7 weeks.	Women who had objective signs of vaginal atrophy, vaginal pH > 5, endometrial thickness < 4 mm as measured by ultrasound, body mass index (BMI) 30 kg/m^2^, and blood pressure < 150/90 mmHg.	Women with 5% superficial cells in the vaginal smears, plasma FSH levels < 40 IU/L, 17b-estradioL levels 70 pmol/l, or malignant changes in the endometrium.	Oxytocin stimulated the growth of the vaginal epithelium cells, so restoring the atrophic vaginal mucosa.
Al-Saqi et al. (2016) [19]	Multicentric, double-blinded, randomized controlled trial.	68	600 IU daily for the first two weeks and 600 IU twice a week for ten weeks.	Placebo.	14 weeks.	Women were healthy > 40-year-old women four years or more after natural menopause. The vaginal mucosa should be atrophic (as evaluated clinically), and atrophy should be verifiable by cytological assessment (superficial cells < 5%), vaginal pH > 5. In addition, plasma follicle-stimulating hormone (FSH) levels should be > 40 IU/L, 17b-estradiol levels < 70 pmol/L, and body mass index (BMI) < 29 kg/m^2^.	Patients were excluded if they used any type of estrogen, including phytoestrogens, herbal products with known estrogenic effect, or hormonal intrauterine device, within three months prior to screening. Exclusion criteria also included a history of prior or current malignant illness or endometrial hyperplasia or having systolic blood pressure > 140 mmHg or diastolic blood pressure > 90 mmHg at screening. Known or suspected drug or alcohol abuse within the12 months prior to screening.	Intravaginal vagitocin increased the percentage of the superficial cells, the maturation value and histological scores of vaginal atrophy.
Torky et al. (2018) [15]	Randomized control trial.	140	1 mg (equivalent to 600 IU).	Placebo.	1 month.	Two years after menopause, suffering from symptoms of vaginal atrophy (vaginal dryness, pain, itching, discomfort, or bleeding with intercourse) and did not use any estrogen or other hormonal treatments (systemic or topical) during the last 4 weeks prior to the study. All women were sexually active.	Women with any serious illnesses, malignancy, or history of malignancy, Women with no sign of vaginal atrophy at the assessment examination were excluded.	Oxytocin gel helped in the restoration of the vaginal epithelium.
Abedi et al. (2020) [17]	Randomized controlled trial.	96	400 IU.	Placebo 400 IU.	8 weeks.	Postmenopausal women aged between 40–50, monogamous with a sexual relationship, whose last menstruation was more than one year prior to the study, and whose score of sexual function was less than 26 according to the Female Sexual Function Index (FSFI).	Women using hormone replacement therapy, having any vaginal bleeding or any breast diseases, using vaginal lubricant, or having any undiagnosed genitalia disorder.	Oxytocin vaginal gel significantly improved vaginal atrophy as well as sexual function.
Jonasson et al. (2020) [16]	Randomized, double-blinded, controlled study.	157	400 IU.	VagiVital (Aqueous Hypromellose-based vaginal gel).	12 weeks.	Females aged 40–65 years who were either postmenopausal or had undergone surgical bilateral oophorectomy, with ≤ 5% superficial cells in vaginal smear cytology, a vaginal pH > 5.0, a body mass index ≤ 32 kg/m2, an endometrial thickness of < 4 mm, and at least one moderate to severe VVA symptom, but who were otherwise in good health and had provided signed informed consent, were considered eligible to participate in the study. In addition, women were to abstain from vaginal sexual activity and the use of vaginal douching within 24 h prior to vaginal pH measurements. Further, women with an intact uterus were required to have an acceptable result from a Pap smear conducted within 6 months prior to the initial dose of study medication.	Women were not permitted to use estrogen alone or estrogen/progestin for any of the following time periods: (a) Vaginal hormonal products (rings, creams, gels, vaginal suppositories) within 12 weeks prior to the screening visit, (b) transdermal estrogen alone or estrogen/progestin products including percutaneous estrogen gels for at least 12 weeks prior to the screening visit, (c) oral estrogen and/or progestin therapy within 12 weeks prior to the screening visit, (d) intrauterine progestin therapy within 12 weeks prior to the screening visit, (e) progestin implants and estrogen alone injectable drug therapy within 12 weeks prior to the screening visit, and (f) estrogen pellet therapy or pregestational injectable drug therapy within 6 months prior to the screening visit.	Significant reductions in the severity of the MBS were seen in both the Aqueous Hypromellose-based vaginal gel and the oxytocin gel groups, but no significant differences in severity reduction were seen between the groups. Both gels were safe and well tolerated.
Zohrabi et al. (2020) [28]	Randomized controlled trial.	96	400 IU.	Placebo.	8 weeks.	Literate women, age 40–60, at least one year passed from their last menstrual period or the level of FSH > 40 IU, monogamous women with a sexual relationship.	Women with vaginal infection, women who used hormone replacement therapy, any undiagnosed genitalia diseases, smokers’ women, a body mass index of more than 30 kg/m^2^, vaginal bleeding or spotting, any breast diseases with unknown cause, using vaginal lubricant at least 15 days before the intervention.	Oxytocin gel improves the vaginal maturation index and subjective symptoms of vaginal atrophy and reduces the pH of the vagina.

These seven eligible papers examined 545 healthy postmenopausal women over the age of 40. There were other inclusion criteria for women to be included in the studies, which were assessed through gynecological examination and cytological evaluations, such as that their last menstruation must be more than one year prior to the study, vaginal pH > 5, body mass index (BMI) < 29 kg/m2, plasma follicle stimulating hormone (FSH) levels > 40 IU/L, 17b-estradiol levels < 70 pmol/L, superficial cells < 5%, endometrial thickness < 4 mm, and a score of sexual function < 26 according to the Female Sexual Function Index (FSFI). Table 2 presents baseline characteristics for the population of the included studies.

**Table 2:** Baseline characteristics for population of the included studies

First author (year)	Group	Sample size	Age (years) Mean (SD)	BMI (kg/m^2^) mean (SD)	Years after menopause mean (SD)
Jonasson et al. (2011) [14]	Oxytocin	10	59.7	27.2	11.2
	Placebo	10	60.4	26	7.4
Al-saqi et al. (2015) [18]	Oxytocin	24	61.1 (5.3)	23.6 (3.2)	NA
	Placebo	16	63.2 (5.8)	24.2 (2.6)	NA
Al-saqi et al. (2015)b [18]	Oxytocin	24	62 (5.7)	23.1 (2.4)	NA
	Placebo	16	63.2 (5.8)	24.2 (2.6)	NA
Al-Saqi et al. (2016) [19]	Oxytocin	33	63 (5.4)	24.7 (1.7)	NA
	Placebo	35	61.3 (7)	23.3 (2.5)	NA
Torky et al. (2018) [15]	Oxytocin	70	54.1 (4.46)	33.45 (4.08)	3.56 (2.11)
	Placebo	70	54.58 (3.41)	33.4 (4.44)	3.2 (1.51)
Abedi et al. (2020) [17]	Oxytocin	44	54.18 (3.31)	28.5 (1.54)	NA
	Placebo	42	54.1 (3.68)	28.8 (1.49)	NA
Jonasson et al. (2020) [16]	Oxytocin	79	58 (3.9)	25.3 (3.5)	NA
	Placebo	78	58.7 (3.1)	25.3 (3.1)	NA
Zohrabi et al. (2020) [28]	Oxytocin	44	54.18 (3.31)	28.5 (1.54)	4.13 (2.01)
	Placebo	42	54.1 (3.68)	28.8 (1.49)	3.78 (2.33)

### Quality of included studies

**Fig. 2:**
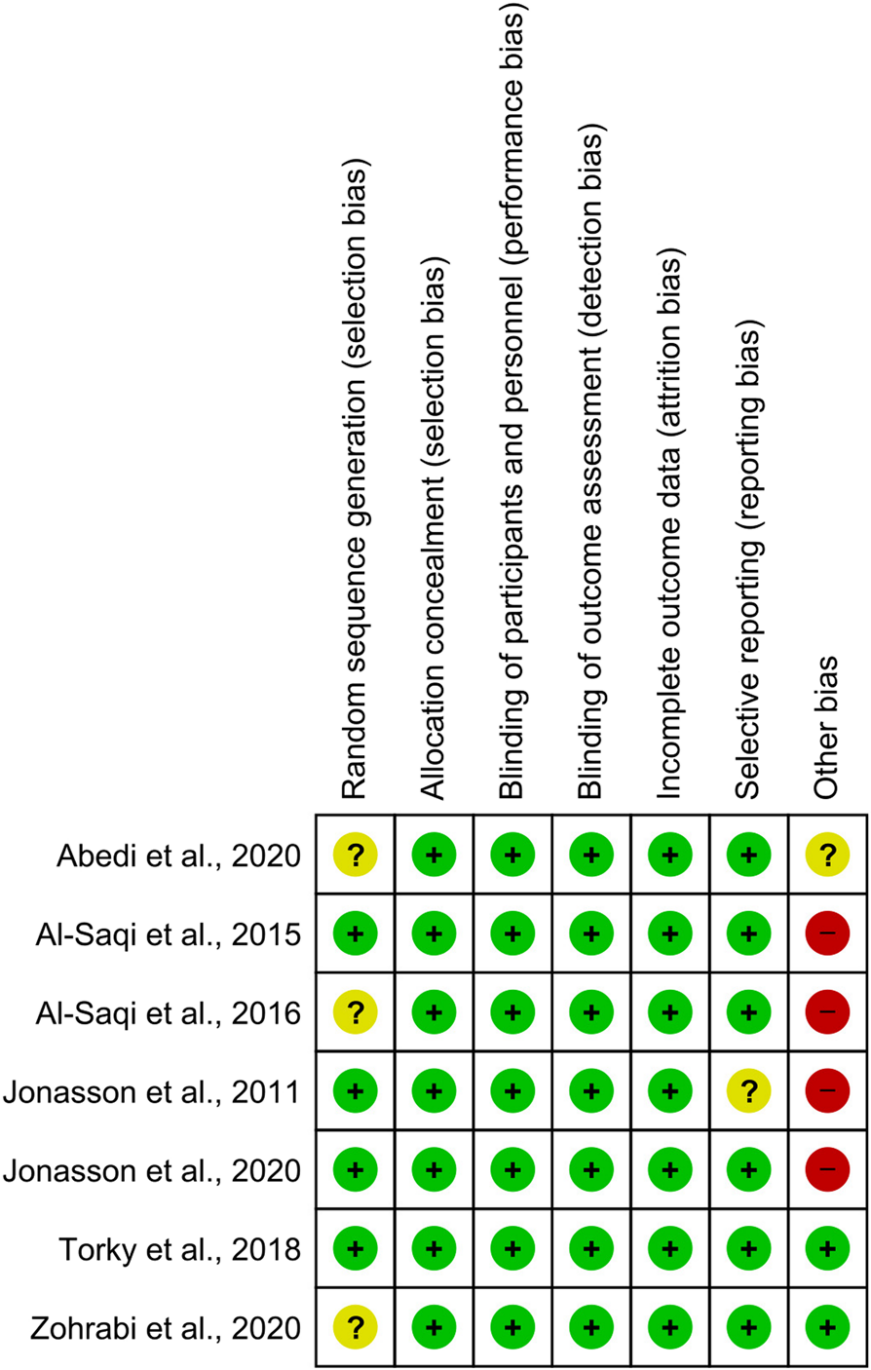
Summary of assessment of the risk of bias

The quality of the included studies was evaluated with the Cochrane Risk of Bias Tool for Randomized Control Trials. The assessment of several sources of bias revealed that all studies had an adequate generation of allocation concealment and appeared to be free from performance, detection, and attrition bias. Three studies [17, 19, 28] were at unclear risk of selection bias related to random sequence generation. The study by Jonasson et al. [14] was at unclear risk of reporting bias. The summary of quality assessment domains is shown in Fig. 2.

### Primary outcome

#### Maturation index

**Fig. 3:**
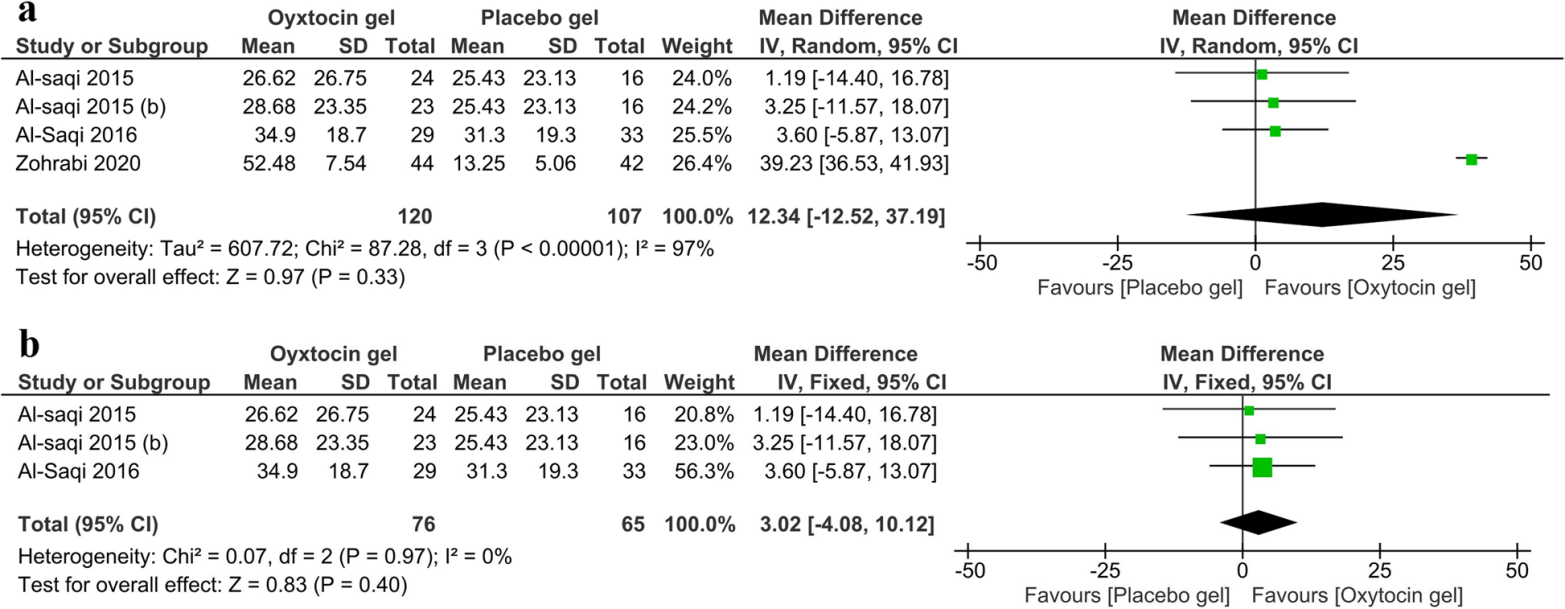
A forest plot shows the mean difference of change in the maturation index (**a**). (**b**) shows the results after excluding Zohrabi et al.

Regarding the maturation index, three trials [18, 19, 28] with a total of 120 patients in the oxytocin arm and 107 patients in the placebo arm were included in the analysis. There was a statistically insignificant increase in the oxytocin arm over the placebo arm (MD = 12.34, 95% CI (-12.52-37.19), *P* = 0.33) (Fig. 3a). The random effect model was used due to significant heterogeneity (I^2^ = 97%, *P* < 0.00001), which became homogenous (I^2^ = 0%, *P* = 0.97) after omitting Zohrabi et al. [28] without significant change in the pooled analysis (MD = 3.02, 95% CI (-4.08-10.12), *P* = 0.4) (Fig. 3b).

### Secondary outcomes

#### Vaginal pH

**Fig. 4:**
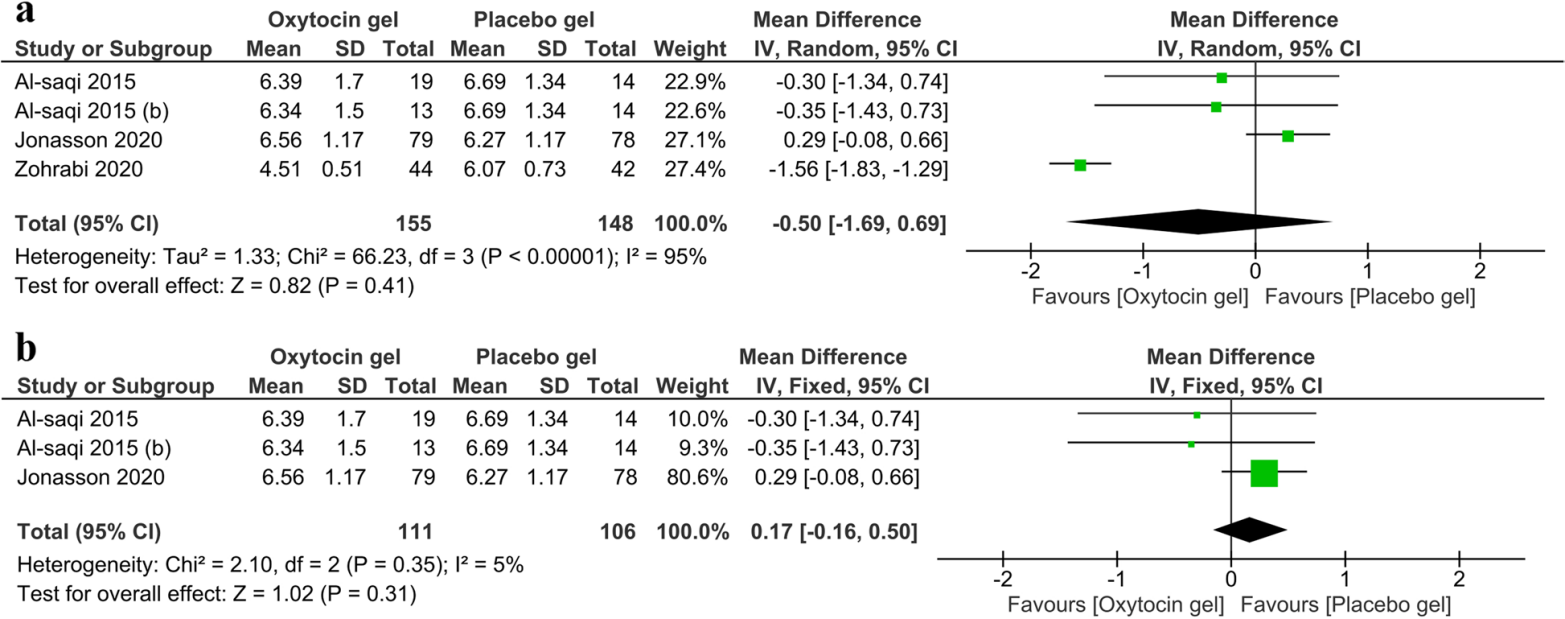
A forest plot shows the mean difference of change in vaginal pH (**a**). (**b**) shows the results after excluding Zohrabi et al.

Regarding vaginal pH, three trials [16, 18, 28] with a total of 155 patients in the oxytocin arm and 148 patients in the placebo arm were included in the analysis. There was a statistically insignificant decrease in the oxytocin arm over the placebo arm (MD = -0.50, 95% CI (-1.69-0.69), *P* = 0.41) (Fig. 4a). The random effect model was used due to significant heterogeneity (I^2^ = 95%, *P* < 0.00001), which became homogenous (I2 = 5%, *P* = 0.35) after omitting Zohrabi et al. [28] without significant change in the pooled analysis (MD = 0.17, 95% CI (-0.16-0.50), *P* = 0.31) (Fig. 4b).

#### Clinically assessed vaginal atrophy

**Fig. 5:**
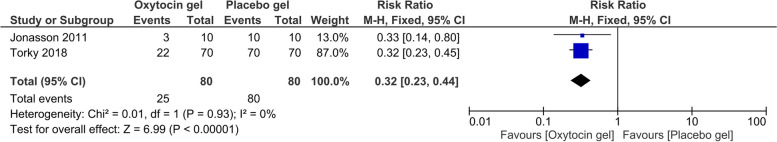
A forest plot shows the risk ratio of patients with vaginal atrophy

Regarding clinically assessed vaginal atrophy, two trials [14, 15] with a total of 80 patients in the oxytocin arm and 80 patients in the placebo arm were included in the analysis. There was a statistically significant decrease in the oxytocin arm over the placebo arm (RR = 0.32, 95% CI (0.23 − 0.10), *P* < 0.00001). The pooled studies were homogenous (I^2^ = 0%, *P* = 0.93) (Fig. 5).

#### Dyspareunia

**Fig. 6:**
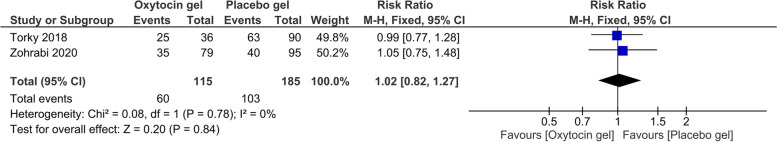
A forest plot shows the risk ratio of patients with dyspareunia

Regarding dyspareunia, two trials [15, 28] with a total of 115 patients in the oxytocin arm and 185 patients in the placebo arm were included in the analysis. There was a statistically insignificant risk in the oxytocin arm over the placebo arm (RR = 1.02, 95% CI (0.82–1.27), *P* = 0.84). The pooled studies were homogenous (I^2^ = 0%, *P* = 0.78) (Fig. 6).

#### Histological evaluation of vaginal atrophy

**Fig. 7:**
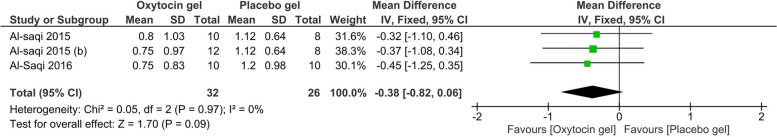
A forest plot shows the mean difference of change in the histological evaluation of vaginal atrophy

Regarding the histological evaluation of vaginal atrophy, two trials [18, 19] with a total of 115 patients in the oxytocin arm and 185 patients in the placebo arm were included in the analysis. There was a statistically insignificant decrease in the oxytocin arm over the placebo arm (MD = -0.38, 95% CI (-0.82-0.06), *P* = 0.09). The pooled studies were homogenous (I^2^ = 0%, *P* = 0.97) (Fig. 7).

#### Safety (endometrial thickness)

**Fig. 8:**
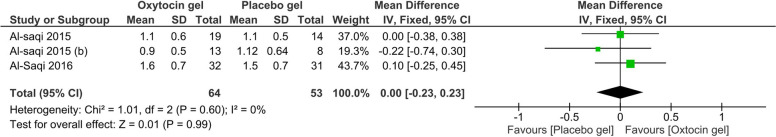
A forest plot shows the mean difference of change in the endometrial thickness

Regarding endometrial thickness, two trials [18, 19] with a total of 64 patients in the oxytocin arm and 53 patients in the placebo arm were included in the analysis. There was a statistically insignificant difference between the two arms (MD = 0.00, 95% CI (-0.23-0.23), *P* = 0.99). The pooled studies were homogenous (I^2^ = 0%, *P* = 0.60) (Fig. 8).

## Discussion

This study was conducted to update the previous systematic review [13] that assessed the efficacy of intravaginal oxytocin gel on the symptoms and laboratory parameters of GSM.

Our meta-analysis showed that the administration of intravaginal oxytocin causes statistically insignificant cytological changes in the vaginal mucosa (vaginal maturation index). On the other hand, Ghorbani and Mirghafourvand [13] documented statistically significant changes because they did not consider the significant heterogeneity. The vaginal maturation index is an indicator of vaginal atrophy that measures the number of intermediate and superficial mucosal cells in relation to immature basal cells [29]. A decreased vaginal maturation index suggests increased vaginal atrophy.

The meta-analysis also showed a statistically insignificant effect of oxytocin on vaginal pH. Four studies, including Jonasson et al. (2020) [14], the newly included study, evaluated changes in vaginal pH, which is considered a valuable parameter in diagnosing vaginal atrophy in addition to the vaginal maturation index. Despite the insignificant changes in these two parameters, the meta-analysis of clinically assessed vaginal atrophy showed oxytocin causing a statistically significant decrease in vaginal atrophy. Clinically assessed vaginal atrophy, as an outcome, was only discussed in two of the old studies [14, 15]. So, the weight of its meta-analysis may not be reliable.

The deficiency of estrogen following menopause causes structural changes in the vaginal epithelium, which can cause the symptoms of GSM, such as itching, dryness, burning, dysuria, and dyspareunia [29]. Despite the importance of dyspareunia as a subjective parameter of vaginal atrophy’s effect on the quality of life of patients with GSM, it was not analyzed in the previous meta-analyses. Furthermore, the meta-analysis of dyspareunia data revealed that vaginal oxytocin does not affect dyspareunia.

Moreover, vaginal atrophy is assessed by histological evaluation, which depends on the number of layers, size of nuclei, and glycogen content of the cells rather than the number of cells in each layer [14]. Despite its high sensitivity in diagnosing vaginal atrophy, histological evaluation is invasive; therefore, it was only done in two studies [18, 19]. The meta-analysis of the histological evaluation showed that the effect of oxytocin was insignificant. The two new trials [16, 28] did not evaluate this outcome; therefore, the results were similar to the previous review. In addition, they did not update the endometrial thickness meta-analysis data, where current evidence suggests that oxytocin does not affect endometrial thickness. Furthermore, we could not perform a meta-analysis of estrogen serum levels because of the difference in measurement units between the studies. Otherwise, these studies [14, 19] found no significant effect of oxytocin on estrogen levels.

The studies included in this systematic review found no statistical evidence of oxytocin affecting either the clinical or lab-based parameters of GSM except for Zohrabi et al. (2020) [28], where a statistically significant effect on vaginal maturation index and vaginal pH was found. However, the same study caused significant heterogeneity.

Hormonal compounds (estrogen alone or estrogen with progesterone) are effective in alleviating the symptoms of GSM, but their safety is questioned [30, 31]. Therefore, it is necessary to find effective alternatives, such as oxytocin. Oxytocin receptors are expressed in various areas, including the vaginal epithelium, where oxytocin plays a role in proliferation and differentiation [32]. In vitro studies and clinical trials [12, 14, 15, 18, 19] have shown that oxytocin has promising results in GSM. In addition, it has protective effects against endometrial, ovarian, and colon cancers [33, 34], unlike estrogen, whose usage is associated with estrogen-dependent cancers [31].

This study answered questions that were not previously discussed, such as whether oxytocin affects different parameters of GSM, such as vaginal pH, vaginal atrophy, and dyspareunia. Moreover, we updated the discussion about the effect of oxytocin on vaginal cytology through the vaginal maturation index by addressing the significant heterogeneity.

Given these findings, the clinical implications of our study indicate that the use of intravaginal oxytocin as a treatment option for GSM should be approached with caution. Although oxytocin has shown promising results in preclinical studies and clinical trials, our study did not provide sufficient evidence to support its clinical efficacy in treating GSM. Clinicians should carefully consider individual patient factors, preferences, and medical history when making treatment decisions for GSM. In cases where patients cannot or prefer not to use hormonal therapy, intravaginal oxytocin may be considered as a potential alternative. However, it is important to acknowledge the limitations of our study, including the small number of trials assessing subjective symptoms and the insignificant results observed in our meta-analysis. Further research is needed to better understand the efficacy and safety profile of oxytocin in GSM management. Future studies should explore optimal dosing regimens, long-term effects, patient satisfaction, and the mechanism of action of oxytocin in GSM. Additionally, larger-scale trials with rigorous study designs and standardized outcome measures are necessary to provide more robust evidence on the clinical effectiveness of oxytocin for GSM. In summary, while our study did not find significant effects of intravaginal oxytocin on GSM symptoms and laboratory parameters, it highlights the need for further investigation. Clinicians should exercise caution and consider personalized treatment approaches for GSM patients, taking into account individual patient factors and preferences, until additional evidence becomes available.

### Strengths and limitations

Our systematic review and meta-analysis have several advantages. We included all available published studies and increased the sample size by 40% over the previous study. We reported important outcomes that had not been evaluated in the previous meta-analysis, including vaginal pH, clinically assessed vaginal atrophy, and dyspareunia. Furthermore, the majority of the outcomes were homogeneous. Finally, whenever high heterogeneity was present, we investigated the robustness of our results using sensitivity analysis and random effect models.

However, the limitations of this study include the small sample size and limited number of studies included in our analyses, short and different follow-up periods, the use of various tools in each study to assess vaginal atrophy, and the use of different drug dosages. Due to data limitations, we were unable to conduct subgroup analyses to investigate the impact of the aforementioned variations. In addition, there was variation in the eligibility criteria of the clinical trials, where some studies [14, 15, 17, 28] used subjective methods such as patient-reported symptoms, whereas other studies [18, 19] used clinical and lab-based criteria for the enrollment of patients.

## Conclusion

The meta-analysis results indicate that vaginal oxytocin does not have a significant effect on genitourinary syndrome of menopause (GSM) patients in terms of the vaginal maturation index, vaginal pH, histological evaluation, endometrial thickness, and dyspareunia. However, a statistically significant decrease was observed in clinically assessed vaginal atrophy. It is important to note that this study’s limitations, including the limited number of included studies and variations in treatment duration, prevent definitive conclusions from being drawn solely based on these findings.

Despite the lack of acceptance of oxytocin as a valuable alternative to estrogen in GSM, the possibility of its efficacy cannot be entirely dismissed. To address the aforementioned limitations, further rigorous clinical trials are strongly encouraged. These studies should aim to provide clearer evidence regarding the impact of oxytocin on different parameters of GSM. Only through additional research can a more comprehensive understanding of oxytocin’s effects in GSM be achieved.

### Supplementary Information

**Additional file 1.**

## Data Availability

All data analyzed during this study are included in this published article or listed in references.

## Abbreviations

BMI: Body mass index
CI: Confidence interval
FSFI: Female Sexual Function Index
FSH: Follicle-stimulating hormone
GSM: Genitourinary syndrome of menopause
IU: International unit
MD: Mean difference
PROSPERO: International Prospective Register of Systematic Reviews
QOL: Quality of life
RCTs: Randomized controlled trials
ROB: Risk of bias
RR: Risk ratio
