# Supplementary material for: The efficacy of oxytocin gel in postmenopausal women with vaginal atrophy: an updated systematic review and meta-analysis
Source: BMC Womens Health. 2023 Sep 16;23:494. doi: 10.1186/s12905-023-02645-0 (PMC10505316; doi:10.1186/s12905-023-02645-0)
Supplement: Supplementary file 1 — Additional file 1. [file 12905_2023_2645_MOESM1_ESM.doc]

Full Search strategy.

| **PubMed/Medline** | |
| --- | --- |
| #1 | "oxytocin"[MeSH Terms] OR "oxytocin"[All Fields] OR "oxytocin s"[All Fields] OR "oxytocine"[All Fields] OR "oxytocins"[All Fields] OR "oxytocin"[MeSH Terms] OR "oxytocin"[All Fields] OR "pitocin"[All Fields] OR "oxytocin s"[All Fields] OR "oxytocine"[All Fields] OR "oxytocins"[All Fields] OR "oxytocin"[MeSH Terms] OR "oxytocin"[All Fields] OR "syntocinon"[All Fields] OR "Vagitocin"[All Fields] OR "Duratocin"[All Fields] OR "carbetocin"[Supplementary Concept] OR "carbetocin"[All Fields] |
| #2 | "intravaginal"[All Fields] OR "intravaginally"[All Fields] OR ("vagina"[MeSH Terms] OR "vagina"[All Fields] OR "vaginal"[All Fields] OR "vaginally"[All Fields] OR "vaginals"[All Fields] OR "vaginitis"[MeSH Terms] OR "vaginitis"[All Fields] OR "vaginitides"[All Fields]) OR ("vulvovaginal"[All Fields] OR "vulvovaginitis"[MeSH Terms] OR "vulvovaginitis"[All Fields]) OR ("dyspareunia"[MeSH Terms] OR "dyspareunia"[All Fields]) OR ("urogenital system"[MeSH Terms] OR ("urogenital"[All Fields] AND "system"[All Fields]) OR "urogenital system"[All Fields] OR "urogenital"[All Fields]) OR "uro-genital"[All Fields] OR ("vulva"[MeSH Terms] OR "vulva"[All Fields] OR "vulvar"[All Fields]) OR "vulvo-vaginal"[All Fields] |
| #3 | "atrophy"[MeSH Terms] OR "atrophy"[All Fields] OR "atrophic"[All Fields] OR "atrophie"[All Fields] OR "atrophy"[MeSH Terms] OR "atrophy"[All Fields] OR "atrophied"[All Fields] OR "atrophies"[All Fields] OR "atrophying"[All Fields] OR "vagina"[MeSH Terms] OR "vagina"[All Fields] OR "vaginal"[All Fields] OR "vaginally"[All Fields] OR "vaginals"[All Fields] OR "vaginitis"[MeSH Terms] OR "vaginitis"[All Fields] OR "vaginitides"[All Fields] OR "vagina"[MeSH Terms] OR "vagina"[All Fields] OR "vaginal"[All Fields] OR "vaginally"[All Fields] OR "vaginals"[All Fields] OR "vaginitis"[MeSH Terms] OR "vaginitis"[All Fields] OR "vaginitides"[All Fields] OR "atrophie"[All Fields] OR "atrophy"[MeSH Terms] OR "atrophy"[All Fields] OR "atrophied"[All Fields] OR "atrophies"[All Fields] OR "atrophying"[All Fields] OR "lubricants"[MeSH Terms] OR "lubricants"[All Fields] OR "lubricant"[All Fields] OR "lubricate"[All Fields] OR "lubricated"[All Fields] OR "lubricates"[All Fields] OR "lubricating"[All Fields] OR "lubrication"[MeSH Terms] OR "lubrication"[All Fields] OR "lubrications"[All Fields] OR "lubricative"[All Fields] OR "lubricity"[All Fields] OR "dryness"[All Fields] |
| #4 | #2 AND #3 |
| #5 | #4 AND #1 |
| **Cochrane library** | |
| #1 | Oxytocin OR Pitocin OR Syntocinon OR Vagitocin OR Duratocin OR Carbetocin |
| #2 | Intravaginal OR vaginal OR Vulvovaginal OR dyspareunia OR urogenital OR uro‐genital OR Vulvar OR vulvo‐vaginal |
| #3 | atrophic OR atrophy OR vaginitides OR vaginitis OR atrophies OR lubrication OR dryness |
| #4 | #2 AND #3 |
| #5 | #1 AND #4 |
| **Web of Science** | |
| #1 | (Oxytocin OR Pitocin OR Syntocinon OR valitocin OR duratoin OR Carbetocin) (Topic) |
| #2 | (Intravaginal OR vaginal OR Vulvovaginal OR dyspareunia OR urogenital OR uro‐genital OR Vulvar OR vulvo‐vaginal) (Topic) |
| #3 | (atrophic OR atrophy OR vaginitises OR vaginitis OR atrophies OR lubrication OR dryness) (Topic) |
| #4 | #2 AND #3 |
| #5 | #1 AND #4 |
| **Scopus** | |
| TITLE-ABS-KEY (((oxytocin OR pitocin OR syntocinon OR vagitocin OR duratocin OR carbetocin) AND ((intravaginal OR vaginal OR vulvovaginal OR dyspareunia OR urogenital OR uro‐genital OR vulvar OR vulvo‐vaginal) AND (atrophic OR atrophy OR vaginitides OR vaginitis OR atrophies OR lubrication OR dryness)))) | |
